# Does the Use of a Checklist Help Medical Students in the Detection of Abnormalities on a Chest Radiograph?

**DOI:** 10.1007/s10278-017-9979-0

**Published:** 2017-05-30

**Authors:** Ellen M. Kok, Abdelrazek Abed, Simon G. F. Robben

**Affiliations:** 1 School of Health Professions Education, Department of Educational Research and Development, Maastricht University, P.O. Box 616, 6200 MD Maastricht, The Netherlands; 2 Faculty of Health, Medicine and Life Sciences, Maastricht University, P.O. Box 616, 6200 MD Maastricht, The Netherlands; 3 Katholisches Karl-Leisner-Klinikum, Albersallee 5-7, 47533 Kleve, Germany; 4 Department of Radiology, Maastricht University Medical Center, P. Debyelaan 25, 6229 HX Maastricht, The Netherlands

**Keywords:** Medical students, Checklist, Chest radiographs, Education, medical, teaching

## Abstract

The interpretation of chest radiographs is a complex task that is prone to diagnostic error, especially for medical students. The aim of this study is to investigate the extent to which medical students benefit from the use of a checklist regarding the detection of abnormalities on a chest radiograph. We developed a checklist based on literature and interviews with experienced thorax radiologists. Forty medical students in the clinical phase assessed 18 chest radiographs during a computer test, either with (*n* = 20) or without (*n* = 20) the checklist. We measured performance and asked participants for feedback using a survey. Participants that used a checklist detected more abnormalities on images with multiple abnormalities (*M* = 50.1%) than participants that could not use a checklist (*M* = 41.9%), *p* = 0.04. The post-experimental survey shows that on average, participants considered the checklist helpful (*M* = 3.25 on a five-point scale), but also time consuming (*M* = 3.30 on a five-point scale). In conclusion, a checklist can help medical students to detect abnormalities in chest radiographs. Moreover, students tend to appreciate the use of a checklist as a helpful tool during the interpretation of a chest radiograph. Therefore, a checklist is a potentially important tool to improve radiology education in the medical curriculum.

## Introduction

Chest radiographs are the most common radiological investigations in hospitals and emergency services [1–3]. Therefore, it is important to give students a strong foundation in chest radiograph interpretation, with the aim of reducing diagnostic errors in the interpretation of a chest radiograph in the future. This is important not only for those students pursuing a career in radiology but also for all medical students. Although radiologists are responsible for the final interpretation of the radiological examinations, many clinicians are the first to view chest radiographs, before the official report is available. All physicians should therefore be able to efficiently and accurately detect and identify potential abnormalities on a chest radiograph [2], for example, in emergency conditions.

However, the topic of radiology is strongly underrepresented in most medical curricula [4–6], and radiology often only serves as an illustration, for example, in anatomy education [7]. Thus, there is a need for efficient methods that can support medical students to miss fewer abnormalities during the interpretation of chest radiographs and can possibly help with the associated diagnostic decision-making. That way, while most medical students will not specialize in radiology, they are at least able to provide a first interpretation of a radiograph that they can act upon.

While widely used, the use of checklists as diagnostic aids for medical students has not been investigated yet [8, 9]. Several authors have argued that checklists are potentially useful instruments to reduce diagnostic errors [8–11], for clinicians of all levels of expertise, but in particular for inexperienced physicians [12]. Safety checklists are broadly used in aviation [8, 13], but also in the operating room [13–15], and in pediatric radiology [16] to improve safety. Ely and colleagues [10] describe three types of checklists: firstly, a general checklist that stimulates physicians to optimize their cognitive approach; secondly, a differential diagnosis checklist to help physicians to prevent missing the most common cause of diagnostic error; and thirdly, a checklist of common pitfalls to optimize the evaluation of pathology.

Checklists are likely to be particularly useful when multiple abnormalities are present in a radiograph [17, 18]. Berbaum and colleagues [19] investigated the effectiveness of checklists for experienced radiologists, with the aim of reducing satisfaction of search errors on chest radiographs. However, they found that this impeded their usual evaluation process. In contrast, medical students might benefit from the use of a checklist because students do not have an automated mental checklist. The effectiveness of checklists to support students in evaluating radiographs has not been investigated so far. The purpose of the current study was to investigate the extent to which medical students benefit from the use of a checklist regarding the detection of abnormalities on a chest radiograph. We evaluated objective effects of a checklist as well as perceived usefulness.

## Materials and Methods

### Research Plan

This study was executed in accordance with the Helsinki Declaration. Prior to data collection, participants were asked to read an information letter and provide informed consent. The participants were then randomized into the checklist group (*n* = 20) or control group (*n* = 20). This randomization process was executed using Research Randomizer (https://www.randomizer.org/). All participants were tested individually or in groups of up to 12 people. The procedure was the same regardless of the number of participants in a session. First, participants were provided with a lecture. Subsequently, they were asked to diagnose 18 chest radiographs during a computer test. They did so using our checklist (checklist group) or without a checklist (control group). Furthermore, we used a survey to investigate the perceived usefulness of the checklist. Finally, participants were thanked for participation and compensated with a 10 euro gift voucher. Ethical approval was obtained from the ethical review board of the Dutch Association for Medical Education (NVMO).

### Participants

In total, 42 master students in medicine (24 female, 18 male) of Maastricht University, the Netherlands, voluntarily participated in our study. All participants were currently going through clinical rotations (i.e., years 4, 5, and 6 of a 6-year program). These participants were recruited by using posters in the Maastricht University buildings. Two male participants were excluded from data analysis because they had received extra-curricular education in radiology and might score significantly higher than other participants. The average age of the other participants was 23.60 years (SD = 1.99). The total group of participants consisted of 11 students (27.5%) from study year 4, 18 participants (45%) from study year 5, and 11 participants (27.5%) from study year 6.

### Materials

#### Checklist

The checklist (see Table 1) consisted of three main parts adapted from the three types of checklists described by Ely and colleagues [10]. Our checklist consisted of an anatomical part, a part with potential pitfalls and a part with frequently missed diagnoses. We hypothesize that this combination will support the students best at detecting abnormalities on chest radiographs. We aimed to keep a balance between a complete checklist with a lot of useful information, and a user-friendly and clear concise checklist.

**Table 1:** The checklist as used in this study

Anatomy	Potential pitfalls	Commonly missed diagnosis
Patient and technique Gender-date-image quality-inspiration position	Wrong patient Wrong data Hypo/hyperinflation	–
Tubes-lines	Tube too deep	–
Pleura-costophrenic angle	Thickening of pleura Pleural effusion	Pneumothorax Hydropneumothorax
Heart-large vessels	Pericardial effusion Silhouette sign	Aneurysm Elongation
Mediastinum-hila-diaphragm Trachea	Lymphadenopathy Foreign body Silhouette sign Tracheal displacement	Pneumomediastinum Pneumoperitoneum Diaphragmatic hernia
Axial skeleton Mandible-vertebrae-ribs	Rib deformities Calcifications	Spinal cord compression Fractures Scoliosis
Appendicular skeleton Clavicles-scapulae-humeri	Tubes, lines, prostheses	Fractures
Soft tissue Abdomen-neck-axilla-chest wall	Nipple shadows	Subcutaneous emphysema Calcifications Surgical clips
Lungs Nodules/masses-lobes-fissures-vascularity	Solitary nodules Opacifications Consolidations	Acute respiratory distress syndrome (ARDS) Pulmonary embolism

The content of the checklist was initially derived from several articles [19, 20] and radiology textbooks [3, 21, 22]. The seven anatomical areas that form the backbone of the checklist (see Table 1) were supplemented with a “potential pitfall” and a “commonly missed diagnosis” section. This initial checklist was discussed during interviews with two senior radiologists, who both have more than 5 years of experience in thoracic imaging. The first version of the checklist was subsequently adapted based on their feedback. All participants were familiar with the anatomic regions and abnormalities covered in the checklist; the radiological appearance of the abnormalities was discussed in the lecture.

Berbaum and colleagues [19] describe that the item “lungs” was preferred as one of the last items of the checklist. Based on that study, we decided to save this item as the last item. For obvious reasons, the first item is checking the patient and technique. Prior research suggests that the particular order of search is less important than sticking to a fixed order [19, 23], so the other items were not put in a specific order.

#### Lecture

Before the actual start of the computer test, all participants were given the same lecture of approximately 15 min. This lecture was a preliminary instruction in which the basic principles of the interpretation of a chest radiograph were covered. In this lecture, radiographs were shown that covered the same pathology as the radiographs used in the computer test. None of the images used in the lecture were used in the computer test. During this lecture, participants also got instructions about the computer test. The lecture was always given by the same instructor with the same slides.

#### Computer Test

All participants completed the same computer test. The computer test was created using Google Forms (Google Drive, https://drive.google.com). The test consisted of 18 conventional AP and PA chest radiographs with the standard question: “abnormalities?” We omitted clinical information since previous studies illustrated that (suggestive) clinical information leads to increased detection of abnormalities compared with irrelevant or no clinical data [24, 25]. Participants were required to type a description of any abnormalities that they detected. They were instructed to describe only the detected abnormalities and no normal findings. If the whole radiograph was considered normal, they were required to type “no abnormalities.” The image was visible while the participants typed their diagnosis. In addition to this computer test, participants were asked demographic questions and finally a survey was conducted with a number of evaluative questions regarding the ease of use and the helpfulness of the checklist.

An experienced radiologist and professor of medical imaging checked all 18 conventional chest radiographs for suitability and difficulty for medical students. The images used had a height of at least 1396 and up to 2500 pixels with a width of at least 1156 and up to 2518 pixels. Since the focus was on detecting abnormalities in images with multiple abnormalities, 13 of the images showed multiple abnormalities (see Table 2). Three images showing a single abnormality and two normal radiographs were included in order to make it not too obvious for participants that often more than one abnormality was visible.

**Table 2:** The distribution of abnormalities over radiographs

Number of abnormalities	Number of chest radiographs
None	2
1	3
2	6
3	5
5	2
Total	18

The images were collected from a teaching file, and all patient information was removed. The participants were asked to evaluate the complete set of radiographs within an hour. This time limit was not only set because of logistical reasons but also to mimic the time pressure in real clinical practice as much as possible.

### Analysis

Participants’ answers were scored by the second author, using a template that was developed by the second and third authors, and blinded for participant group. The participants could earn one point per detected abnormality. Half a point was awarded when participants reported only half of the abnormality, for example, when a student answers “silhouette sign right heart contour” if there was a silhouette sign of both heart contours. Separate analyses were conducted for normal images (*n* = 2), images with a single abnormality (*n* = 3) and images with multiple abnormalities (*n* = 13). Data was not normally distributed, so Mann-Whitney *U* tests were conducted, with a significance level of *α* = 0.05. We used *r* as an effect size, which is the *Z*-score divided by the square root of *N*. 0.3 is considered a medium effect and 0.5 is a large effect. Data analysis was performed using SPSS version 20 (IBM, Redmond).

#### Post-Experimental Questionnaire

After the test, we asked the participating students in the checklist group three questions to evaluate the ease of use and the helpfulness of our checklist (see Table 3). The participants in the control group were asked one question related to the potential use of a checklist (see Table 3). Finally, all participants were asked to write down what they considered to be an advantage and a disadvantage of a checklist.

**Table 3:** Student feedback regarding the post-experiment survey

Question	Answer options	Frequency (*n* = 20)	Mean score (*n* = 20)
Q1 To what extent did you find the use of a checklist time consuming?	0 = not at all 1 2 3 4 5 = very much	*n* = 0 (0%) *n* = 2 (10%) *n* = 3 (15%) *n* = 7 (35%) *n* = 4 (20%) *n* = 4 (20%)	3.25 (1–5)
Q2 To what extent has the checklist helped you in judging the chest radiographs?	0 = not at all 1 2 3 4 5 = very much	*n* = 0 (0%) *n* = 1 (5%) *n* = 4 (20%) *n* = 4 (20%) *n* = 10 (50%) *n* = 2 (10%)	3.30 (1–5)
Q3 Would you ever use this checklist during the assessment of a chest X-radiograph in clinical practice?	1. no 2. yes, sometimes 3. yes 4. yes, always	*n* = 5 (25%) *n* = 1 (5%) *n* = 12 (60%) *n* = 2 (10%)	2.55 (1–4)
Q4 Would you have preferred to use a checklist during assessment of a chest radiograph in clinical practice?	1. no 2. yes, sometimes 3. yes, always	*n* = 0 (0%) *n* = 9 (45%) *n* = 11 (55%)	2.55 (1–3)

Data are presented as mean (interquartile range) or *n* (%). *Q* = question. Q1, Q2, and Q3 were posed to the participants in the checklist group; Q4 was posed in the control group

## Results

Table 4 provides an overview of descriptive statistics for the two groups and three types of images. For images with multiple abnormalities, a medium-sized, significant difference was found, *U* = 124.5, *Z* = −2.04, *p* = 0.04, *r* = −0.32; participants in the checklist group identified significantly more abnormalities than participants in the control group. For the normal images, no significant difference in mean ranking was found between the checklist group and the control group, *U* = 179.5, *Z* = −0.61, *p* = 0.54, *r* = −0.10. No significant difference between the two groups was found for images with a single abnormality, *U* = 194.0, *Z* = −0.17, *p* = 0.87, *r* = −0.03.

**Table 4:** Average percentage correct and standard deviations for the two groups and three types of images

	Normal images		Single abnormality		Multiple abnormalities	
	*M*	SD	*M*	SD	*M*	SD
Checklist group	37.5%	31.9	27.5%	30.7	50.1%	12.2
Control group	32.5%	37.3	25.0%	27.3	41.9%	13.0

*M* mean, *SD* standard deviation

The three evaluative questions that were asked to the checklist group are shown in Table 3. Participants experienced benefit from the checklist; they considered it to be a helpful tool, but the checklist was also experienced as being time consuming. Only five participants (25%) say that they would not use the checklist in clinical practice; the other 75% of the participants report a preference for using a checklist in practice at least sometimes. The great advantage of the checklist that was mentioned frequently (*n* = 13, 65%) was that the checklist functioned as a mnemonic. A large number of participants (*n* = 9, 45%) mentioned that the checklist was complete. However, an almost equivalent number of participants (*n* = 7, 35%) claimed that the checklist was incomplete. These participants mentioned that the incomplete checklist might put the assessor on a false trail. According to many participants (*n* = 8, 40%), the main disadvantage that they experienced was that the use of the checklist is time consuming. Again, some participants (*n* = 4, 20%) claimed also that the checklist should be more concise, while others (*n* = 4, 20%) preferred the checklist to be more extensive. All participants from the control group indicated that they would have preferred to have a checklist (see Table 3).

## Discussion

We evaluated the effectiveness of a checklist for chest radiograph interpretation by medical students, as well as the perceived usefulness. Overall, our study showed that the use of a checklist by medical students led to a significant increase in the detection of abnormalities on a conventional chest radiographs with multiple abnormalities present. The effect size was moderate; on average, the checklist helped detect more than three (*M* = 3.2) additional abnormalities per 13 radiographs. That is one additional abnormality per four radiographs. This is a valuable and clinically relevant effect. Although several possible improvements are mentioned, in general, participants were positive about the checklist. Therefore, we can conclude that a checklist is a potentially effective method to support medical students in the interpretation of a chest radiograph with the aim of increasing detection of abnormalities.

An important aspect of checklist use is time pressure. Participants mentioned that the checklist is time consuming. Therefore, there seems to be a mismatch between the time consuming use of the checklist and the limited time available at the clinic. This is an important paradox regarding our checklist and checklist use in general. The checklist should contain enough useful information. However, it must be also concise and clear. In the current experiment, the checklist was used to improve the identification of abnormalities. A checklist could also be used as a learning method. When used as a learning method, the time-consuming use in the initial phase is acceptable, because we expect that students will eventually be more efficient while using our checklist, leading to a normal workflow, because they automate the checklist.

Another important paradox was the large group of participants (45%) that mentioned that the checklist was complete while almost an equivalent group of participants (35%) remarked that the checklist was incomplete. The participants who considered the checklist to be incomplete argued that due to missing information, the checklist might put the assessor on a false trail. For example, when the checklist includes the item “rib fractures,” participants might forget other pathology involving the ribs or fractures related to other bone structures. It is therefore extremely important to help students realize that the checklist is a useful tool, but not a panacea. It could be helpful, for example, to provide possibilities for students to practice checklist use by working through annotated cases in a teaching file (27), so they learn to value the advantages and disadvantages of the checklist.

One of our consultant thorax radiologists suggested including a final item in the checklist: “seek help” (i.e., consult a senior staff member or the radiologist on duty). For the purpose of this experiment, it was not possible to include this item as participants were required to finish the test individually. However, during clinical practice, medical students or young doctors always have the opportunity to consult with their own supervisor or with a radiologist (on duty). We therefore suggest that checklists used for medical students in the clinical situation should include this item, so they stimulate students to ask for help when needed.

The current study has several limitations. Checklist compliance could not be tested; we only measured the outcome of checklist use, and not the process. This makes it unclear how the checklists were used. For example, the checklist could be used during the interpretation process, or only afterwards to check the diagnosis. Sibbald and colleagues, for example, found that verifying a diagnosis with a checklist resulted in improved diagnostic accuracy in cardiopulmonary medicine, but only if the checklist could be used to re-examine the diagnostic information after an initial diagnosis was given [26]. Furthermore, when abnormalities are not reported, this can be either be due to a miss, a failure to see an abnormality, or due to a failure to correctly diagnose it [20]. The current setup did not allow for a differentiation between those two errors, and further research should monitor the process of checklist use in order to investigate how the checklist is used, and what type of errors are avoided.

Another limitation of this study is the lack of longitudinal follow-up. Although the checklist was useful for supporting chest radiograph interpretation in this single-session test, we do not know whether prolonged checklist use might support learning. A checklist might support students in acquiring a systematic approach to radiograph interpretation [27]. Further research should investigate the long-term effects of checklist use for learning chest radiograph interpretation.

Finally, the current study focused on improving radiograph interpretation when multiple abnormalities are present and thus included a small number of radiographs with no abnormalities or a single abnormality. Further research with a larger amount of cases from each group is required to investigate whether checklists only impact performance when multiple abnormalities are present. Also, we only investigated the effectiveness of checklist use for fourth-, fifth-, and sixth-year medical students, and not for other potential users of checklists. Many radiologists do actually use checklists in clinical practice. Structured reporting, for example, is thought to have effects similar to the use of a checklist [28]. Further research should investigate the generalizability of our findings to other groups.

## Conclusions

Radiology is strongly underrepresented in medical curricula, while a large proportion of clinicians will have to interpret radiographs at some point in clinical practice. While checklists have to balance completeness and conciseness, our checklists was generally considered valuable. Our checklist helped medical students to detect more than three extra abnormalities per 13 radiographs in cases with multiple abnormalities.
